# Supermarket purchase contributes to nutrition-related non-communicable diseases in urban Kenya

**DOI:** 10.1371/journal.pone.0185148

**Published:** 2017-09-21

**Authors:** Kathrin M. Demmler, Stephan Klasen, Jonathan M. Nzuma, Matin Qaim

**Affiliations:** 1 Department of Agricultural Economics and Rural Development, University of Goettingen, Goettingen, Germany; 2 Department of Economics, University of Goettingen, Goettingen, Germany; 3 Department of Agricultural Economics, University of Nairobi, Nairobi, Kenya; SOAS, University of London, UNITED KINGDOM

## Abstract

**Background:**

While undernutrition and related infectious diseases are still pervasive in many developing countries, the prevalence of non-communicable diseases (NCD), typically associated with high body mass index (BMI), is rapidly rising. The fast spread of supermarkets and related shifts in diets were identified as possible factors contributing to overweight and obesity in developing countries. Potential effects of supermarkets on people’s health have not been analyzed up till now.

**Objective:**

This study investigates the effects of purchasing food in supermarkets on people’s BMI, as well as on health indicators such as fasting blood glucose (FBG), blood pressure (BP), and the metabolic syndrome.

**Design:**

This study uses cross-section observational data from urban Kenya. Demographic, anthropometric, and bio-medical data were collected from 550 randomly selected adults. Purchasing food in supermarkets is defined as a binary variable that takes a value of one if any food was purchased in supermarkets during the last 30 days. In a robustness check, the share of food purchased in supermarkets is defined as a continuous variable. Instrumental variable regressions are applied to control for confounding factors and establish causality.

**Results:**

Purchasing food in supermarkets contributes to higher BMI (+ 1.8 kg/m^2^) (*P*<0.01) and an increased probability (+ 20 percentage points) of being overweight or obese (*P*<0.01). Purchasing food in supermarkets also contributes to higher levels of FBG (+ 0.3 mmol/L) (*P*<0.01) and a higher likelihood (+ 16 percentage points) of suffering from pre-diabetes (*P*<0.01) and the metabolic syndrome (+ 7 percentage points) (*P*<0.01). Effects on BP could not be observed.

***Conclusion*s:**

Supermarkets and their food sales strategies seem to have direct effects on people’s health. In addition to increasing overweight and obesity, supermarkets contribute to nutrition-related NCDs. Effects of supermarkets on nutrition and health can mainly be ascribed to changes in the composition of people’s food choices.

## Introduction

While undernutrition and related infectious diseases are still widespread problems in many developing countries [1], overweight, obesity, and nutrition-related non-communicable diseases (NR-NCD) are growing epidemically [2–5]. Seventy-five percent of all people with diabetes live in developing countries [6,7]. Africa has the world’s highest prevalence of hypertension [8]. Almost three-quarters of all worldwide NCD-related deaths occur in low-income and middle-income countries [5]. These problems will likely grow further in the years and decades to come [9,10], also because most developing countries have little experience with diagnosing, treating, and preventing NCDs [11–13]. NCDs are placing a substantial economic and social burden on countries in terms of human suffering, increased health care costs, and reduced labor productivity [14,15].

It is widely known that “unhealthy” diets and physical inactivity contribute to overweight and obesity and hence a higher prevalence of NR-NCDs [16]. Depending on the stage of transition in a given society, changes in lifestyle and eating habits lead to an increased intake of processed foods, saturated and total fats, salt, sugar, and caloric beverages [17–20]. The globalization of agri-food systems, with its rapid spread of supermarkets in developing countries, may contribute to the observed nutrition transition and thus also to overweight, obesity, and related NR-NCDs [21–24]. In this study, we analyze possible links between the spread of supermarkets, people’s body mass index (BMI), and several other indicators of NR-NCDs.

What type of diets people consume and where they buy their food depends on their income, education, lifestyles, and various other socioeconomic factors. However, the food retail environment and the accessibility to different types of markets and shops can also play important roles [21,25]. Modernization in the food retail sector is typically associated with changes in the types of foods offered, prices, packaging sizes, and shopping atmosphere. Especially in urban areas of developing countries, consumers increasingly buy their food in supermarkets instead of wet markets or other traditional retail outlets [25–28]. Except for a few large supermarket stores in big cities, where fresh foods are also offered, many supermarket chains in developing countries primarily concentrate on selling processed foods, especially when they open up new stores in smaller towns [29,30].

Recent research revealed significant associations between supermarket purchase and dietary shifts in different developing countries [29,31–35]. While the concrete results differ and depend on the particular context, several studies showed that people buying in supermarkets tend to consume more energy and a higher share of processed foods [20,29,31,33]. The consumption of highly processed food is often associated with higher overweight and obesity [36,37]. Studies carried out in Guatemala and Kenya suggested indeed that purchasing food in supermarkets tends to increase BMI and the likelihood of overweight and obesity, even after controlling for income and other possible confounding factors [31,32]. We are not aware of any study that went beyond nutritional status and analyzed possible links between supermarkets and NR-NCDs. Better understanding possible health implications of the rapid spread of supermarkets could help in designing food and nutrition policies aimed at curbing the epidemic of NR-NCDs.

We contribute to the literature by investigating the effects of purchasing food in supermarkets on nutrition and health in Kenya. Kenya has experienced a rapid growth of supermarkets in recent years [29]. The share of national grocery sales through supermarkets in Kenya is about 10%; when only focusing on larger cities the share is already much higher [38]. Kenya is still struggling with relatively high rates of child undernutrition. At the same time, NR-NCDs are growing problems. More than 26% of all adults in Kenya are either overweight or obese [39]. The national prevalence of diabetes and hypertension is estimated at 2.5% and 35%, respectively [7,40].

For this study, we collected data on food purchase and consumption behavior, other socioeconomic characteristics, nutrition, and health from randomly selected adults in urban areas of Central Kenya. We use regression models to estimate the effects of supermarket purchase on BMI, blood glucose, pre-diabetes, blood pressure, pre-hypertension, and the metabolic syndrome. Since BMI and the prevalence of NCDs can also be influenced by factors other than supermarket purchase, it is important to control for such confounding factors in the statistical analysis. We employ an instrumental variable (IV) approach, which helps to reduce endogeneity bias and establish causality with observational data.

## Materials and methods

### Ethics statement

This study was approved by the Ethics Commission of the University Medical Center Goettingen (http://www.ethikkommission.med.uni-goettingen.de/; study ID 25/9/14) and the Ethics and Research Committee of the Kenyatta National Hospital in Nairobi (http://erc.uonbi.ac.ke; study ID P192/04/2015). Written consent was obtained from each study participant.

### Study design

This study uses cross-sectional data collected in 2015 from households and individual household members in three small towns in Central Kenya. A focus on small towns was chosen because some of these towns already have a supermarket, while others have not. The three towns, Ol Kalou and Njabini in Nyandarua County and Mwea in Kirinyaga County, where purposively selected due to their supermarket characteristics. In Kenya, as in other developing countries, supermarket chains started their business in the big cities, now they are also expanding to smaller towns [29]. Ol Kalou has had a supermarket already since 2002 and Mwea since 2011. Njabini did not yet have a supermarket in 2015, although there were concrete plans to open one in the near future and the building was already constructed. Beyond having or not having a supermarket, the three towns are similar in terms of size, ethnic structure of the population, infrastructure conditions, and financial and social institutions [41]. This setup provides a quasi-experimental setting, allowing the comparison of consumers with varying degrees of supermarket exposure.

The sampling strategy for this study builds on an earlier household survey that was conducted in the same three towns in 2012 [29,32,42]. In each town, households for inclusion were selected using systematic random sampling. Since recent census data were not available, population statistics and the help of local administrators were used. First, all neighborhoods (residential estates) were listed in each town. Then, for each neighborhood, household lists were compiled, from which households were selected randomly. To obtain a representative sample at town level and avoid clustering, households were selected from all neighborhoods. The 2012 data were collected to analyze the effects of supermarkets on consumers’ diets and nutrition. Health indicators to analyze effects on NR-NCDs were not collected in 2012, but were added to the survey in 2015.

The 2015 data, which are used in this study, were collected between May and July 2015. The survey comprised 433 randomly selected households. In these households, interviews were conducted and measurements were taken from 550 male and female adult household members above 18 years of age. The interviews were conducted in local languages (Kikuyu, Kiswahili, and English). All measurements, including weight, height, waist- and hip circumference, blood pressure, and fasting blood glucose, were taken by experienced local nurses, which were trained according to standards of anthropometric measurements by the Centers for Disease Control and Prevention [43].

Interviews and measurements took place in participants’ homes. Each household was visited twice. During the first visit, the interviews were conducted and appointments made for the second visit, during which measurements were taken. The second visits took place a few days later during early morning hours, as participants had to be fasting for the blood glucose measurements. In some cases, it was not possible to take fasting measurements. For the analysis of fasting blood glucose, pre-diabetes, and the metabolic syndrome only 496 adults from 400 households could be used, as non-fasting measurements had to be dropped. The means of key variables between the full sample and the smaller subsample were compared, without finding significant differences. About 5% of the randomly selected women were pregnant. We carried out all analyses with and without including pregnant women. As results were very similar in terms of directions and magnitudes, we decided to keep pregnant women in the sample, as the larger number of observations adds to statistical efficiency.

Power calculations showed that the sample with 550 observations, observed effect sizes, and a significance criterion of 95%, yields statistical power ranging between 0.88 and 0.97 for the different nutrition and health indicators, thus exceeding common standards for adequacy.

### Data

Body weight measurements were taken from all adult individuals with an accuracy of 0.1 kg in minimum clothing and without shoes on a digital scale (range: 10–150 kg). Height was measured with portable stadiometers (SECA; range: 20–205 cm) with accuracy of 0.7 cm while standing upright, barefoot, and without headgear according to international standards [43,44]. BMI was calculated from the body weight and height (BMI = body weight in kg / body height in meters squared) and classified according to WHO criteria [45].

Fasting blood glucose (FBG), which is an indicator of diabetes, was determined through one capillary blood drop using the finger prick procedure. Diabetes and pre-diabetes were defined according to criteria by the American Diabetes Association: a person was classified as being diabetic or pre-diabetic if his/her FBG exceeded 7.0 mmol/L or 5.6 mmol/L, respectively [46]. Systolic blood pressure (SBP) and diastolic blood pressure (DBP) were determined by using a digital auscultatory blood pressure cuff. A SBP ≥ 140 mmHg or a DBP ≥ 90 mmHg were defined as hypertensive state; a SBP ≥ 120 mmHg and a DBP ≥ 80 mmHg were defined as pre-hypertensive state [8]. The metabolic syndrome (MetS) was defined according to the classifications of the International Diabetes Federation [47]. As triglyceride levels and high-density-lipoprotein cholesterols were not measured, a person was classified as suffering from MetS when the following three conditions were all fulfilled: central obesity (waist circumference males ≥ 94 cm; females ≥ 80 cm), raised FBG (≥ 5.6 mmol/L), and raised blood pressure (SBP ≥ 130 mmHg; DBP ≥ 85 mmHg).

Food purchase and consumption decisions were captured through a 30-day food consumption recall at the household level. The person responsible for food purchases and food preparation was asked which of the 176 foods and drinks listed in the questionnaire had been consumed by any household member during the 30 days prior to the interview. Respondents were also asked to specify the quantities consumed of each food item, the source (supermarket, wet market, small shop, own production etc.), and the price. Household expenditures for non-food goods and services were also captured during the interviews. Total per capita consumption expenditures for food and non-food goods and services were used to measure household living standards. In the development economics literature, consumption expenditures are generally considered a more reliable indicator of living standards than income [29].

### Statistical methods

All statistical analyses were conducted using Stata version 13 (StataCorp, College Station, Texas). The unit of analysis is the individual adult. At first, mean values of the nutrition and health outcome variables of interest are compared between individuals in households that did and did not buy food items in supermarkets. Buying in supermarkets means that at least some of the food items consumed during the 30 days prior to the survey were obtained from a supermarket. Not buying in supermarkets means that all of the food items consumed were obtained from traditional retail outlets or other sources. The nutrition and health outcomes considered for individual *i* (*NH*_*i*_) are BMI (kg/m^2^), FBG (mmol/L), SBP (mmHg), and DBP (mmHg), all measured as continuous variables. In addition, being classified as overweight/obese, pre-diabetic (including pre-diabetes and diabetes), pre-hypertensive (including pre-hypertension and hypertension), and suffering from MetS is captured through binary outcome variables.

Simple comparisons between households with and without supermarket purchase can provide a first impression of possible nutrition and health effects, but they should not be overinterpreted because observed differences in outcomes may also be caused by other factors. To control for possible confounding factors and estimate net effects of purchasing in supermarkets, regression models of the following type are estimated:

$${NH}_{i}=\beta_{0}+\beta_{1}\;S_{j}+\beta_{2}X_{ij}+u_{ij}$$ (1)

where *S*_*j*_ is the binary “treatment” variable defined as 1 if household *j* (in which individual *i* lives) purchased food items in a supermarket and 0 otherwise. ***X***_*ij*_ is a vector of individual and household characteristics, including age, education, sex, living standard, and levels of physical activity, among others. *u*_*ij*_ is a random error term.

As individuals and households decide themselves whether or not they purchase food in supermarkets, *S*_*j*_ is likely endogenous. In particular, *S*_*j*_ may be correlated with unobserved characteristics that could themselves have an effect on nutrition and health outcomes. Such a correlation could lead to selection bias (or omitted variable bias) in the estimation of the treatment effect, *β*_1_. For instance, unobserved lifestyle factors could potentially cause such bias. To reduce selection bias and other possible problems of endogeneity, an instrumental variable approach is applied [48,49].

#### Instrumental variable approach

The interpretation of causal effects with cross-section, observational data is possible when using an instrumental variable (IV) approach [50]. The IV approach helps to overcome problems of endogeneity with the treatment variable by replacing the potentially endogenous variable with predicted values, using one or more valid instruments in a two-stage estimation procedure. IV models are widely used in applied economics [51–53], but also in the nutrition and public health literature [32,54,55]. An instrument is valid if it is exogenous, correlated with the treatment variable, and uncorrelated with all outcome variables [48]. Previous studies that analyzed the effect of supermarket purchase on food choices and nutrition had used distance to the nearest supermarket as an instrument [29,31,32]. The same instrument is also employed here. Distance to the nearest supermarket from each individual home was measured through Global Positioning System (GPS) coordinates.

While the placement of supermarkets is not a random process, the decision is made by supermarket owners based on criteria that cannot be influenced by individual consumers. Both towns with a supermarket (Ol Kalou and Mwea) only had one supermarket, which was located in the town center, where many other shops were also found. Hence, the location of supermarkets was exogenously determined and not linked to socioeconomic characteristics of a particular neighborhood within the town. In order to double-check this assumption we used data from Njabini, the town where no supermarket had opened until 2015, and computed the correlation between supermarket purchase (some households in Njabini use supermarkets in other towns) and distance to the town center of Njabini (exactly the point where the building for the new supermarket was constructed). The correlation was insignificant (*r* = 0.03; *P*>0.10).

Distance to the nearest supermarket is closely correlated with supermarket purchase (*r* = 0.67). S1 Table in the Supporting Material also confirms that distance to the nearest supermarket is highly significant in the first stage regression of the IV model, passing the test for a strong instrument. To examine whether distance to supermarket is correlated with any of the nutrition and health outcomes through mechanisms other than supermarket purchase, we used a simple test by additionally including the instrument in the set of models described in Eq (1). While not being a standard overidentification test, this approach is widely used in the literature to evaluate the plausibility of the exclusion restriction when only one instrument is available [56,57]. Test results are shown in S2 and S3 Tables in the Supporting Material. Supermarket distance was not statistically significant in any of these models (*P*>0.10). Hence, distance to supermarket seems to fulfill all requirements for a valid instrument.

The IV models are specified as follows:

$$S_{j}=\propto_{0}+\propto_{1}D_{j}+\propto_{2}X_{ij}+\varepsilon_{ij}$$ (2)

$${NH}_{i}=\delta_{0}+\delta_{1}{\hat{S}}_{j}+\delta_{2}X_{ij}+\omega_{ij}$$ (3)

Eq (2) is the first stage selection equation, whereas Eq (3) is the outcome equation. *D*_*j*_ is the instrument, distance to the nearest supermarket measured in km. ${\hat{S}}_{j}$ is the instrumented treatment variable resulting from predictions based on the selection equation. Thus, *δ*_1_ can be interpreted as the unbiased treatment effect. *ε*_*ij*_ and *ω*_*ij*_ are random error terms. The other variables are defined as above. These models were estimated with Stata IV estimators. For the binary outcome variables, a linear probability IV specification was used. For comparison, ordinary least-squares (OLS) estimators without instrumental variable were also employed. In all models, standard errors are cluster-corrected at town level to avoid problems of heteroskedasticity.

#### Robustness checks

Several tests are used to check how robust the estimation results are to variations in model specifications or changes in some of the other underlying assumptions. A first test relates to the models with binary outcome variables. Instead of the linear probability specifications that we use in the main part of the analysis, we re-run the models with standard probit and IV probit specifications, in order to see whether the estimated effects change.

A second test relates to the definition of purchasing food in supermarkets as treatment variable. In the main analysis, we use a binary treatment variable that takes a value of 1 if the household purchased any food in a supermarket during the last 30 days and 0 otherwise. However, supermarket users typically also use traditional retail outlets, meaning that they only purchase parts of their total food in supermarkets. If supermarkets affect people’s diets, nutrition, and health, we would expect that the effects increase with higher shares of food purchased in supermarkets. Such a dose dependency is tested by using a continuous treatment variable “share of supermarket purchase”, defined as the percentage share of supermarket food expenditures in total household food expenditures during the last 30 days.

A third test relates to the assumptions in the IV modeling approach. IV models are a common statistical tool to reduce endogeneity bias and establish causality in impact evaluations with observational data. However, the reliability of results depends on the validity of the instrument, which is hard to prove beyond any possible doubt. An alternative approach to reduce issues of endogeneity without the need for an instrument is to use a statistical differencing technique with individual fixed effects [48]. This requires panel data. While we do not have panel data for the health outcomes of interest, we do have panel data for the socioeconomic and nutrition variables by combining the 2015 survey with the data collected in 2012 in the same three towns [29,32]. The sample in 2012 and 2015 was not identical, but there was a significant overlap in households and individuals, so that panel data models can be estimated. We use a panel data model for BMI with fixed effects and random effects specifications to check the robustness of the IV results. The advantage of the fixed effects specification is that any time-invariant heterogeneity at individual, household, or town level, whether observed or unobserved, is properly controlled for.

## Results

Out of all 550 study participants, more than half (292) lived in households that purchased food in supermarkets; the rest (258) lived in households that did not buy any food in supermarkets during the 30 days prior to the survey. Descriptive statistics and definitions for the nutrition and health outcomes and the explanatory variables used in the analysis are shown in Table 1.

**Table 1:** Descriptive statistics for adults in households that buy and do not buy food in supermarkets.

Variable	Definition	All	Does not buy in SM	Buys in SM
Body mass index	Body mass index in kg/m^2^	25.99 (5.23)	25.15 (4.92)	26.74*** (5.38)
Underweight	= 1 if BMI (in kg/m^2^) < 18.5	0.04 (0.20)	0.04 (0.20)	0.04 (0.19)
Overweight	= 1 if BMI (in kg/m^2^) ≥ 25.0 and < 30.0	0.32 (0.47)	0.26 (0.44)	0.36** (0.48)
Obese	= 1 if BMI (in kg/m^2^) ≥ 30.0	0.22 (0.41)	0.18 (0.39)	0.25* (0.43)
Overweight/obese	= 1 if BMI (in kg/m^2^) ≥ 25.0	0.53 (0.50)	0.45 (0.50)	0.61*** (0.49)
Fasting blood glucose ^a^	Fasting blood glucose in mmol/L	5.04 (1.37)	4.99 (1.54)	5.07 (1.20)
Pre-diabetic ^a^	= 1 if FBG (in mmol/L) ≥ 5.6	0.15 (0.36)	0.10 (0.30)	0.20*** (0.40)
Diabetic ^a^	= 1 if FBG (in mmol/L) ≥ 7.0	0.03 (0.18)	0.03 (0.18)	0.03 (0.18)
Systolic blood pressure	Systolic blood pressure in mmHg	132.42 (21.57)	134.54 (23.69)	130.54** (19.35)
Diastolic blood pressure	Diastolic blood pressure in mmHg	86.65 (13.06)	87.48 (14.02)	85.91 (12.13)
Pre-hypertensive	= 1 if SBP/DBP (in mmHg) ≥ 120 / ≥ 80	0.82 (0.38)	0.83 (0.38)	0.82 (0.39)
Hypertensive	= 1 if SBP/DBP (in mmHg) ≥ 140 / ≥ 90	0.41 (0.49)	0.43 (0.50)	0.39 (0.49)
Metabolic syndrome ^a^	= 1 if all 3 of the following criteria are fulfilled: waist circumference (in cm) for F/M > 80 / > 94; SBP/DBP (in mmHg) ≥ 130 / ≥ 85; FBG (in mmol/L) ≥ 5.6	0.07 (0.26)	0.06 (0.23)	0.08 (0.28)
Share of supermarket purchase (%)	Share of total household food expenditures from food purchases in supermarkets within the last 30d	7.25 (11.01)	0.00 (0.00)	13.65*** (11.88)
Expenditure per capita	Total (food and non-food) expenditures per capita of the last 30 d in 1000 Kenyan shilling	14.16 (9.34)	11.70 (7.36)	16.33*** (10.32)
Education	School education in years of attendance	9.67 (3.49)	8.72 (3.61)	10.52*** (3.14)
Intensive work	Physical effort demanded for work within the last 7 d (self-estimated on a scale 1–4) multiplied by typical amount of work (considering occupational activities within the last 6 mo) in h/wk	123.02 (77.35)	124.47 (85.32)	121.74 (69.68)
Physical activity	All leisure time physical activity (including walking) within the last 30 d in h/wk	15.98 (11.06)	16.85 (11.24)	15.21* (10.86)
Distance to hospital	Distance to nearest district hospital from home ^b^, in km	10.57 (7.09)	12.82 (3.92)	8.57*** (8.53)
Age	Age in y	38.10 (12.29)	40.18 (14.09)	36.26*** (10.11)
Female	= 1 if being female	0.75 (0.43)	0.71 (0.46)	0.79** (0.41)
Married	= 1 if being married	0.75 (0.43)	0.73 (0.45)	0.76 (0.43)
Household size	Count of all household members that were either household head or ≥ 180 d present in the household within the last 365 d	4.45 (1.97)	4.79 (2.29)	4.15*** (1.58)
History diabetes	= 1 if either mother, father, grandparents or siblings suffer(ed) from diabetes type 2	0.21 (0.41)	0.20 (0.40)	0.22 (0.42)
History heart attack	= 1 if either mother, father, grandparents or siblings suffer(ed) from heart attack before the age of 60 y	0.06 (0.23)	0.06 (0.24)	0.05 (0.23)
History diabetes/heart attack	= 1 if either history diabetes or history heart attack is 1	0.25 (0.43)	0.24 (0.43)	0.25 (0.44)
Smoking	= 1 if smoked > 0 cigarettes/cigars within the last 30 d	0.05 (0.22)	0.06 (0.23)	0.04 (0.21)
Distance to supermarket	Distance to the nearest supermarket ^b^ including the town without supermarket where the next supermarket was not in the same town, in km	16.29 (21.48)	31.54 (21.24)	2.82*** (9.14)
Number of observations	Number of adults(>18 y) included in the analysis	550	258	292

Values are means with SD in parentheses.

^a^ Limited sample size n = 496 with non-supermarket buyers (n = 230) and supermarket buyers (n = 266).

^b^ Measured through GPS coordinates. DBP, diastolic blood pressure; FBG, fasting blood glucose; GPS, Global Positioning System; KES, Kenyan shilling; n, number of observations; SBP, systolic blood pressure; SM, supermarket.

* Difference between those shopping and not shopping in supermarkets is significant at 10% level

** Difference between those shopping and not shopping in supermarkets is significant at 5% level

*** Difference between those shopping and not shopping in supermarkets is significant at 1% level.

Mean BMI is significantly higher among those that purchased food in supermarkets. Similarly, prevalence rates of overweight and obesity are also significantly higher among individuals that purchased food in supermarkets. For the health variables, the comparison is more mixed. While supermarket buyers are more likely to be pre-diabetic, they have lower mean blood pressure levels than non-supermarket buyers. For the other health indicators, no significant differences between the two groups can be observed.

### Supermarket effects on nutrition and health

Tables 2 and 3 provide results of the IV model estimates for the continuous and binary nutrition and health outcome variables. Looking at Table 2, statistically significant effects of purchasing food in supermarkets on BMI and FBG can be seen. After controlling for confounding factors, purchasing food in supermarkets increases BMI by 1.82 kg/m^2^ and FBG by 0.30 mmol/L.

**Table 2:** Regression results for the effects of supermarkets on BMI, fasting blood glucose, systolic and diastolic blood pressure.

	BMI (kg/m^2^)	FBG (mmol/L)	SBP (mmHg)	DBP (mmHg)
Buys in supermarket	1.82*** (0.24)	0.30*** (0.06)	1.98 (1.33)	1.23 (0.86)
Expenditure per capita	0.11*** (0.02)	0.01*** (0.00)	-0.03 (0.05)	0.03 (0.04)
Education, y	-0.00 (0.10)	-0.01 (0.01)	-0.42*** (0.14)	-0.21** (0.10)
Intensive work, h/wk	0.01** (0.00)	0.00 (0.00)	0.00 (0.01)	-0.00 (0.00)
Physical activity, h/wk	-0.02** (0.01)	0.00 (0.00)	-0.01 (0.02)	-0.01 (0.01)
Age, y	0.11*** (0.03)	0.02*** (0.00)	0.88*** (0.02)	0.41*** (0.02)
Distance to hospital, km	0.05*** (0.00)	0.02*** (0.00)	-0.09 (0.10)	0.01 (0.07)
Female	3.59*** (0.28)	0.20** (0.09)	-4.84** (2.31)	-2.81** (1.39)
Married	1.01** (0.45)	-0.11 (0.13)	-0.04 (1.41)	0.56 (0.51)
Household size	-0.12*** (0.04)	-0.01 (0.04)	-1.21*** (0.25)	-0.54*** (0.09)
Smoking	-2.14*** (0.65)	-0.17 (0.14)	-12.57*** (1.40)	-7.30*** (1.78)
History diabetes		0.26* (0.14)		
History heart attack			-0.08 (0.36)	-0.49 (1.94)
Constant	15.31*** (2.15)	3.46*** (0.19)	112.80*** (5.62)	76.73*** (2.92)
R-squared	0.23	0.07	0.28	0.17
Number of observations	550	496	550	550

Coefficient estimates of instrumental variable (IV) models are shown with standard errors in parentheses. Standard errors are cluster-corrected at town level. “Distance to nearest supermarket” was used as instrument for “buys in supermarket”. BMI, body mass index; DBP, diastolic blood pressure; FBG, fasting blood glucose; SBP, systolic blood pressure.

* Significant at 10% level

** Significant at 5% level

*** Significant at 1% level.

**Table 3:** Regression results for the effects of supermarkets on the probability of being overweight/obese, pre-diabetic, pre-hypertensive, and suffering from metabolic syndrome.

	Overweight/Obese	Pre-diabetic	Pre-hypertensive	MetS
Buys in supermarket	0.204*** (0.02)	0.164*** (0.01)	-0.014 (0.02)	0.068*** (0.01)
Expenditure per capita	0.008*** (0.00)	0.001 (0.00)	-0.000 (0.00)	0.000 (0.00)
Education, y	0.014* (0.01)	-0.001 (0.00)	-0.001 (0.00)	-0.006** (0.00)
Intensive work, h/wk	0.001** (0.00)	0.000 (0.00)	-0.000 (0.00)	0.000 (0.00)
Physical activity, h/wk	-0.001 (0.00)	0.001 (0.00)	0.001 (0.00)	0.000 (0.00)
Age, y	0.010*** (0.00)	0.006*** (0.00)	0.006*** (0.00)	0.005*** (0.00)
Distance to hospital, km	0.005*** (0.00)	0.001* (0.00)	-0.003*** (0.00)	0.001*** (0.00)
Female	0.258*** (0.04)	0.008 (0.01)	-0.050*** (0.02)	0.017 (0.02)
Married	0.077 (0.05)	0.021*** (0.01)	-0.034** (0.02)	0.041 (0.03)
Household size	-0.005 (0.01)	0.004 (0.01)	-0.013 (0.01)	-0.001 (0.00)
Smoking	-0.204*** (0.03)	0.034*** (0.01)	-0.002 (0.03)	-0.050*** (0.02)
History diabetes		0.096** (0.04)		
History heart attack			0.105*** (0.03)	
History diabetes/heart attack				0.071*** (0.01)
Constant	-0.537*** (0.16)	-0.289** (0.12)	0.776*** (0.04)	-0.172*** (0.03)
R-squared	0.18	0.07	0.05	0.08
Number of observations	550	496	550	496

Coefficient estimates of instrumental variable (IV) linear probability models are shown with standard errors in parentheses. Standard errors are cluster-corrected at town level. “Distance to nearest supermarket” was used as instrument for “buys in supermarket”. Overweight/obese: BMI ≥ 25 kg/m^2^; Pre-diabetic: FBG (in mmol/L) ≥ 5.6 (also includes diabetic with FBG ≥ 7.0); Pre-hypertensive: SBP/DBP (in mmHg) ≥ 120/80 (also includes hypertensive with SBP/DBP ≥ 140/90); Metabolic syndrome (MetS): defined through three parameters: waist circumference (in cm) F/M > 80 /94 plus SBP/DBP (in mmHg) ≥ 130/85 and FBG (in mmol/L) ≥ 5.6. DBP, diastolic blood pressure; FBG, fasting blood glucose; MetS, metabolic syndrome; SBP, systolic blood pressure

* Significant at 10% level

** Significant at 5% level

*** Significant at 1% level.

These effects are further underlined by the results in Table 3, showing that purchasing food in supermarkets increases the prevalence of overweight and obesity, pre-diabetes, and MetS. Buying food in a supermarket increases the likelihood of overweight/obesity by 20 percentage points, the likelihood of being pre-diabetic by 16 percentage points, and the likelihood of suffering from MetS by 7 percentage points, holding all other factors constant. For comparison, OLS estimates of the same models are shown in S4 and S5 Tables in the Supporting Material.

### Other factors influencing nutrition and health outcomes

Looking at the socioeconomic control variables in Tables 2 and 3, it can be seen that household per capita expenditure, which is used to measure living standards, has a significantly positive effect on BMI, as well as on the likelihood of being overweight or obese. Similarly, positive effects on BMI and overweight/obesity are found for being female and being married. Holding other factors constant, female adults have a 3.6 kg/m^2^ higher BMI and are 26 percentage points more likely to be overweight/obese than male adults. Being female is also positively related with FBG, but negatively related with blood pressure. Smoking is negatively related with BMI and overweight/obesity, but also with blood pressure, which is rather unexpected as smoking was identified as one of the major contributors to any coronary heart diseases [8]. It should be mentioned that the number of self-reported smokers in our sample is very small; the negative association of smoking with blood pressure may possibly be due to measurement error and/or unobserved lifestyle factors. Family histories of diabetes and heart attack are positively associated with the likelihood of suffering from pre-diabetes, pre-hypertension, and MetS. Age is positively associated with all nutrition and health outcomes, implying that older people are more likely to be overweight/obese and to suffer from NR-NCDs.

### Robustness checks

Standard probit and IV probit specifications for the models with binary outcome variables are shown in S6 Table in the Supporting Material. These alternative estimates lead to similar results as the linear probability models in Table 3.

The results with the continuous treatment variable “share of supermarket purchase” are summarized in Tables 4 and 5 (full results are shown in S7 and S8 Tables). These alternative estimates confirm the general findings obtained with the binary treatment variable: the signs and significance levels of the treatment effects are identical to those in Tables 2 and 3. A one percentage point increase in the share of food purchased in supermarkets leads to a 0.15 kg/m^2^ higher BMI and a 0.02 mmol/L increase in fasting blood glucose (Table 4). Similarly, a one percentage point increase in the share of food purchased in supermarkets raises the probability of being overweight/obese by 1.6 percentage points, the probability of being pre-diabetic by 1.3 percentage points, and the probability of suffering from MetS by 0.5 percentage points (Table 5). It should be stressed that for many households in the sample the share of supermarket purchase is still quite low (14% on average). The continuous treatment effects are point estimates, which should not be extrapolated linearly over wide variations of the treatment variable. Nevertheless, the estimates clearly suggest that there is a dose dependency. We also estimated alternative models with the continuous treatment variable, but only using the subsample of supermarket users. These alternative models yielded results that are very similar to the full-sample results in Tables 4 and 5.

**Table 4:** Regression results for the effects of supermarket purchase (%) on BMI, fasting blood glucose, systolic and diastolic blood pressure.

	BMI (kg/m^2^)	FBG (mmol/L)	SBP (mmHg)	DBP (mmHg)
Share of supermarket purchase, %	0.15*** (0.02)	0.02*** (0.00)	0.16 (0.11)	0.10 (0.07)
Constant	14.22*** (2.18)	3.30*** (0.21)	111.61*** (6.34)	75.99*** (3.32)
Number of observations	550	496	550	550

Coefficient estimates of instrumental variable (IV) models are shown with standard errors in parentheses. Standard errors are cluster-corrected at town level. “Distance to nearest supermarket” was used as instrument for “share of supermarket purchase”. Control variables are not shown for brevity. Full results are provided in S7 Table. BMI, body mass index; DBP, diastolic blood pressure; FBG, fasting blood glucose; SBP, systolic blood pressure.

* Significant at 10% level

** Significant at 5% level

*** Significant at 1% level.

**Table 5:** Regression results for the effects of supermarket purchase (%) on the probability of being overweight/obese, pre-diabetic, pre-hypertensive, and suffering from metabolic syndrome.

	Overweight/Obese	Pre-diabetic	Pre-hypertensive	MetS
Share of supermarket purchase, %	0.016*** (0.00)	0.013*** (0.00)	-0.001 (0.00)	0.005*** (0.00)
Constant	-0.660*** (0.16)	-0.379*** (0.13)	0.784*** (0.05)	-0.209*** (0.03)
Number of observations	550	496	550	496

Coefficient estimates of instrumental variable (IV) linear probability models are shown with standard errors in parentheses. Standard errors are cluster-corrected at town level. “Distance to nearest supermarket” was used as instrument for “share of supermarket purchase”. Control variables are not shown for brevity. Full results are provided in S8 Table. MetS, metabolic syndrome.

* Significant at 10% level

** Significant at 5% level

*** Significant at 1% level.

As explained, in a final robustness check we used a panel data model for BMI to estimate the effect of supermarket purchase without the need for an instrument. Fixed effects and random effects specifications of this panel data model confirm a positive and significant effect of supermarket purchase on BMI (S9 Table). These robustness checks suggest that the general findings are not driven by a particular type of model specification, by the definition of the treatment variables, the choice of instrument, or unobserved lifestyle factors.

## Discussion

### Study limitations

We have analyzed the effects of purchasing food in supermarkets on NR-NCDs among urban adults in Kenya. The methodological approach used has a few limitations. First, the observational data are cross-section in nature, which complicates the identification of causal effects. We used an IV modeling approach to control for confounding factors and reduce possible issues of endogeneity. For BMI, the effects were also confirmed with a panel data model, but for the health outcomes no panel data were available. Repeated collection of data for all relevant outcome variables through additional survey rounds would help to further test the robustness of the estimation results. Second, and related to the previous point, classifying health status based on single measurements can be imprecise, especially for health outcomes such as diabetes or hypertension. Employing well-trained and experienced nurses, using reliable clinical instruments, and taking all measurements at the same time of the day, as done in this study, can reduce sources of imprecision, but not completely. Third, due to budget constraints we were only able to collect certain health indicators and not others that could have been useful as well. For instance, the classification of MetS here was based on only three factors, instead of five that are commonly used [58]. Only considering three factors may lead to an underestimation of the true number of people suffering from MetS. Fourth, data were only collected in three towns. While these three towns are typical for medium-sized urban municipalities in Central Kenya, the sample is not representative for the country as a whole.

### Rising rates of nutrition-related non-communicable diseases

In spite of the mentioned limitations, the results contribute to the literature because this is the first study that has attempted to analyze the effects of the spread of supermarkets on NR-NCDs in developing countries. In Kenya, as in many other developing countries, rapidly rising prevalence rates of obesity and NR-NCDs are observed, so that a better understanding of causes and contributing factors is important from public health and policy perspectives. In the study region in Central Kenya, mean BMI among adults was 26.0 kg/m^2^ during the survey in 2015. The 2012 data collected in the same three towns showed a mean BMI of 24.9 kg/m^2^ [32]. Hence, mean BMI increased considerably within a period of only three years. Similarly, between 2012 and 2015 the prevalence of overweight has increased from 27% to 32%, and the prevalence of obesity from 14% to 22%.

A study with data collected in 2010 in Nairobi reported a prevalence of hypertension of 23% [59], compared to a prevalence of hypertension of 41% in the 2015 sample used here. Furthermore, 15% of the individuals in the sample used here suffered from pre-diabetes and 7% from MetS in 2015. Our estimated prevalence of pre-diabetes is higher than other available estimates for Kenya: according to the 2015 estimates of the International Diabetes Federation (IDF), the national prevalence of pre-diabetes in Kenya is 9.5% [7]. While we do not claim to have nationally representative data, our higher prevalence of 15% may still be more realistic. For most developing countries, IDF statistics are based on estimates and extrapolations using doctors’ records rather than data from representative samples [7,60]. Doctors’ records may underestimate the prevalence of NR-NCDs, because many people in developing countries do not see a doctor on a regular basis.

### Summary of supermarket effects

The regression results suggest that the spread of supermarkets contributes to rising body weight. Buying food in supermarkets instead of (or in addition to) traditional retail outlets was shown to increase BMI by 1.82 kg/m^2^, after controlling for confounding factors. Relatedly, supermarket purchase increases the likelihood of being overweight or obese by 20 percentage points. The directions and the magnitudes of these results are consistent with earlier studies carried out in Kenya and Guatemala [31,32]. The analysis also revealed that buying food in supermarkets increases FBG by 0.30 mmol/L and the likelihood of being pre-diabetic and suffering from MetS by 16 and 7 percentage points, respectively. The general findings were also confirmed in a robustness check using the share of supermarket food purchases as a continuous treatment variable. We found no evidence that buying in supermarkets increases BP or the likelihood of suffering from pre-hypertension. The insignificant effect on hypertension might be due to the multifactorial character of this medical condition, which is not yet well examined, especially not in Africa.

Even though our results are consistent with the literature, the estimated effects in our study (for nutrition and health outcomes) as well as in previous studies (confined to nutrition outcomes) are relatively large in magnitude. Since all the results derive from cross-sectional data, one should be careful not to over-interpret the precision of the estimates. However, regardless of the exact magnitude of effects, the estimates and robustness checks depict a clear tendency, namely that supermarkets influence consumers’ nutrition and health, also after controlling for other relevant socioeconomic and lifestyle factors.

### Expected mechanisms of supermarket effects

The observed effects of supermarkets on nutrition and health can be explained by changing food offers and shopping environments that influence consumer choices and diets. Supermarkets in developing countries tend to offer different types of foods than wet markets and other traditional retail outlets. Levels of processing, packaging sizes, and prices are often different as well. Previous research has shown that people who buy in supermarkets consume more calories and a higher share of processed foods [21,23,29,31–33]. And energy-dense, processed foods and beverages are known to contribute to overweight and obesity [10,17,18].

**Fig 1:**
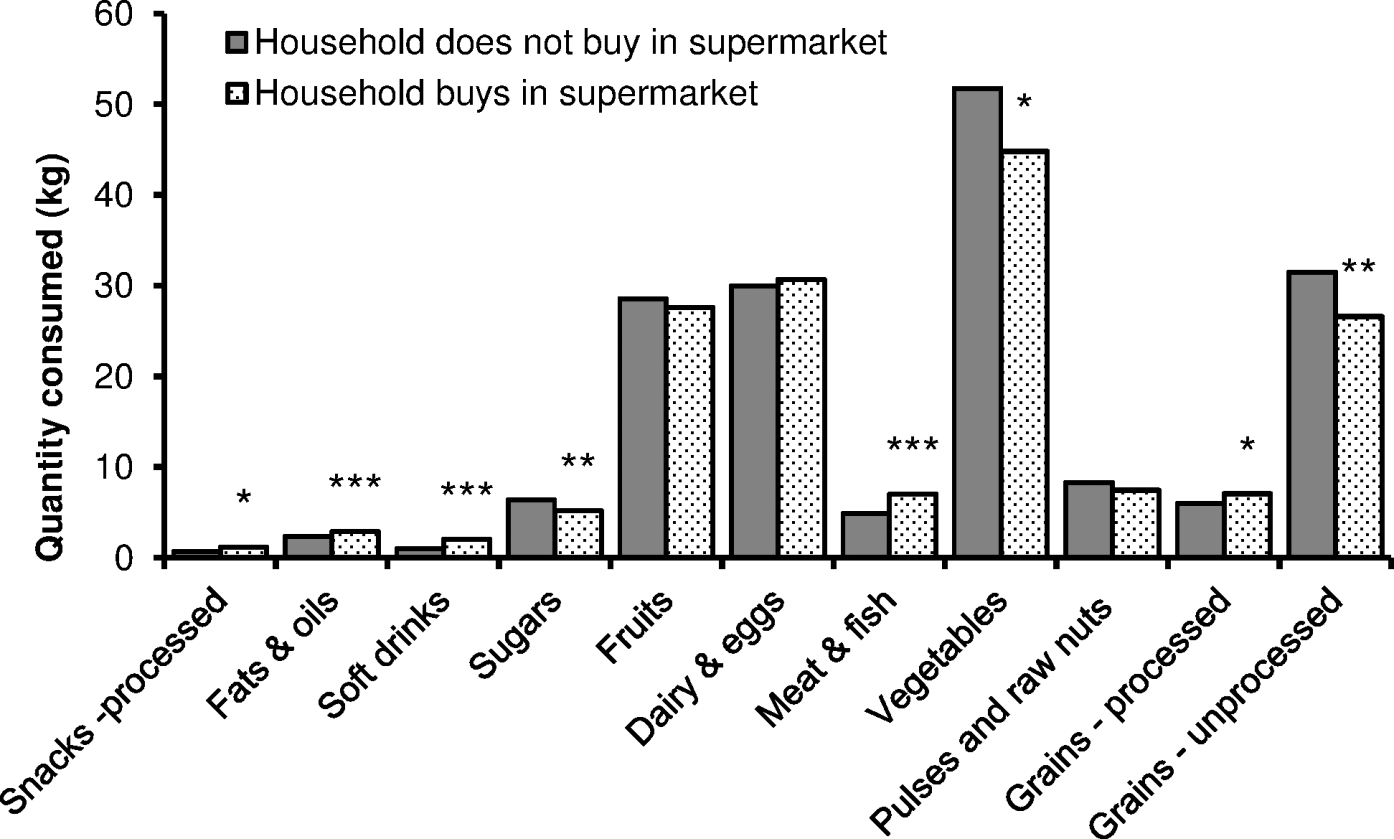
Comparison of mean food consumption within last 30d in households that buy and do not buy food in supermarkets (n = 433). *Mean is different at *P* < 0.10; ** Mean is different at *P* < 0.05; *** Mean is different at *P* < 0.01.

These general relationships are also true in Kenya. Fig 1 shows differences in dietary patterns between households that buy and do not buy food in supermarkets. The observed differences in the consumption of various food groups are not very large, which is due to the fact that most of the households so far only buy part of their total foods consumed in supermarkets. Nevertheless, many of the differences are statistically significant. Households that purchase food in supermarkets consume higher quantities of processed snacks, fats and oils, soft drinks, meat and fish, and processed grains. On the other hand, they consume significantly lower quantities of vegetables and unprocessed grains. These differences in diets may contribute to increased overweight and obesity among supermarket buyers and thus also to a higher prevalence of NR-NCDs.

That such differences in diets are likely caused by supermarkets and their particular food offers was shown in another recent study with data from Kenya [42]. Demmler et al. [42] confirmed that supermarkets contribute to increased consumption of highly processed foods, meats, dairy, and vegetable oils. They also showed that supermarkets decrease the amounts of energy obtained from unprocessed food items such as fresh vegetables and grains. While traditional retailers also sell processed foods, the processed food items purchased in supermarkets seem to be of additional nature. That is, supermarket users purchase additional quantities of processed foods without necessarily reducing processed food purchases from traditional shops. This may possibly be explained by supermarkets selling popular brands or larger packaging sizes that are not available in traditional shops. Also pricing and advertising strategies and the self-service character of supermarkets may incentivize consumers to use supermarkets and buy additional quantities [42].

We expect that most of the effects of supermarkets on NR-NCDs are channeled through higher BMI. However, there are also other possible mechanisms. One other possible mechanism is the reduced amount of bioactive compounds in “supermarket” diets that contain lower quantities of vegetables and unprocessed foods. There is evidence that bioactive compounds–including phytochemicals, vitamins, minerals, and fibers–can reduce the risk of diabetes and other chronic diseases even after controlling for BMI [61].

### Policy implications

Results of this study suggest that the rapid spread of supermarkets contributes to the nutrition transition and the rising epidemic of NR-NCDs in developing countries. However, this does not mean that supermarkets should be prohibited, as they may also have positive effects for public health and development. Compared to traditional food markets in developing countries, supermarket supply chains are often more efficient, which can make food more accessible for poor population segments [21,25,32]. Recent studies showed that supermarkets can contribute to reduced rates of child undernutrition in some situations [32,62]. Food quality, food diversity, and food safety may also be higher in supermarkets than in traditional markets [30,35,63]. Finally, studies have shown that small-scale farmers in developing countries may benefit from participating in newly emerging supermarket supply chains [26,28]. Against this background, it will be important for policymakers to strengthen the positive aspects of supermarket growth, while reducing negative implications to the extent possible. A critical aspect is to shape food environments that allow and instigate consumers to make more healthy food choices. This may require broader awareness building and education towards healthy nutrition, as well as appropriate regulation. For instance, outside of the big cities, supermarkets in developing countries often only sell processed foods. Requiring or supporting supermarkets to also offer fresh fruits and vegetables, and to position such a fresh produce section in a key place within the store, could be one possible route for nutrition-sensitive policymaking.

## Conclusion

This study suggests that buying food in supermarkets increases BMI, fasting blood glucose, and the probability of being overweight/obese, pre-diabetic, and suffering from the metabolic syndrome. Since supermarket users consume larger quantities of highly processed and energy-dense foods, we reckon that the nutrition and health effects are mainly driven by supermarkets influencing people’s dietary choices. This would mean that the rapid spread of supermarkets in developing countries directly contributes to the nutrition transition. However, premature judgements should be avoided, as supermarkets can also have positive effects for public health and development. We have highlighted new aspects and dimensions of the effects of supermarkets on nutrition and health in developing countries. This is a new research direction where the available evidence is still relatively thin. Given the rapidly rising prevalence of NR-NCDs in many developing countries, more research on the role of changing food environments and appropriate policy responses that account for the complexity of effects will be needed.

## Supporting information

S1 Table First stage results of instrumental variable model. (PDF)

S2 Table Validity test of instrument in models for continuous nutrition and health outcomes. (PDF)

S3 Table Validity test of instrument in models for binary nutrition and health outcomes. (PDF)

S4 Table Regression results for the effects of supermarkets on BMI, fasting blood glucose, systolic and diastolic blood pressure comparing OLS and IV estimations. (PDF)

S5 Table Regression results for the effects of supermarkets on the probability of being overweight/obese, pre-diabetic, pre-hypertensive, and suffering from metabolic syndrome comparing OLS and IV estimations. (PDF)

S6 Table Regression results for the effects of supermarkets on the probability of being overweight/obese, pre-diabetic, pre-hypertensive, and suffering from metabolic syndrome comparing probit and IV probit estimations. (PDF)

S7 Table Full regression results for the effects of supermarket purchase (%) on BMI, fasting blood glucose, systolic and diastolic blood pressure. (PDF)

S8 Table Full regression results for the effects of supermarket purchase (%) on the probability of being overweight/obese, pre-diabetic, pre-hypertensive, and suffering from metabolic syndrome. (PDF)

S9 Table Regression results for the effects of supermarkets on BMI with panel data model. (PDF)

S1 Data File Data set on household and individual characteristics of 550 adults from urban Kenya. (XLS)
