# Supplementary material for: Supermarket purchase contributes to nutrition-related non-communicable diseases in urban Kenya
Source: PLoS One. 2017 Sep 21;12(9):e0185148. doi: 10.1371/journal.pone.0185148 (PMC5608323; doi:10.1371/journal.pone.0185148)
Supplement: S1 Table — (PDF) [file pone.0185148.s001.pdf]

S1 Table. First stage results of instrumental variable model

	Buys in supermarket
Distance to supermarket, km	-0.014*** (0.001)
Expenditure per capita	0.009** (0.004)
Education, y	0.011*** (0.001)
Intensive work, h/wk	-0.000*** (0.000)
Physical activity, h/wk	0.000 (0.001)
Age, y	-0.002 (0.001)
Distance to hospital, km	-0.009** (0.004)
Female	0.040*** (0.005)
Married	0.047 (0.054)
Household size	0.002 (0.005)
Smoking	0.004 (0.033)
Constant	0.656*** (0.108)
R-squared	0.52
F-statistic	123.51
Number of observations	550

Notes: First stage of instrumental variable estimation (selection equation), where "distance to nearest supermarket" is used as an instrument for "buys in supermarket". Coefficient estimates are shown with robust standard errors in parentheses. * Significant at 10% level; ** Significant at 5% level; *** Significant at 1% level.
