# Supplementary material for: Supermarket purchase contributes to nutrition-related non-communicable diseases in urban Kenya
Source: PLoS One. 2017 Sep 21;12(9):e0185148. doi: 10.1371/journal.pone.0185148 (PMC5608323; doi:10.1371/journal.pone.0185148)
Supplement: S2 Table — (PDF) [file pone.0185148.s002.pdf]

S2 Table. Validity test of instrument in models for continuous nutrition and health outcomes

	BMI (kg/m^2)	FBG (mmol/L)	SBP (mmHg)	DBP (mmHg)
Buys in supermarket	0.71 (0.57)	0.07 (0.22)	-3.28 (2.44)	-1.19 (1.58)
Distance to supermarket, km	-0.02 (0.01)	-0.00 (0.00)	-0.07 (0.05)	-0.03 (0.04)
Constant	16.04*** (1.39)	3.60*** (0.30)	116.22*** (6.04)	78.30*** (3.85)
R-squared	0.23	0.08	0.28	0.18
Number of observations	550	496	550	550

Notes: Coefficients are shown with robust standard errors in parentheses. Not all control variables are shown for brevity. Included control variables are the same as in all other models: expenditure, education, intensive work, physical activity, age, distance to hospital, being female, being married, household size, smoking, history of diabetes, and history of heart attack. DBP, diastolic blood pressure; FBG, fasting blood glucose; SBP, systolic blood pressure. * Significant at 10% level; ** Significant at 5% level; *** Significant at 1% level.
