# Supplementary material for: Supermarket purchase contributes to nutrition-related non-communicable diseases in urban Kenya
Source: PLoS One. 2017 Sep 21;12(9):e0185148. doi: 10.1371/journal.pone.0185148 (PMC5608323; doi:10.1371/journal.pone.0185148)
Supplement: S3 Table — (PDF) [file pone.0185148.s003.pdf]

S3 Table. Validity test of instrument in models for binary nutrition and health outcomes

	Overweight/obese	Pre-diabetic	Pre-hypertensive	MetS
Buys in supermarket	0.062 (0.06)	0.068 (0.04)	0.024 (0.06)	0.035 (0.03)
Distance to supermarket, km	-0.002 (0.00)	-0.001 (0.00)	0.001 (0.00)	-0.000 (0.00)
Constant	-0.444*** (0.14)	-0.228* (0.12)	0.470*** (0.16)	-0.152 (0.09)
R-squared	0.19	0.08	0.05	0.08
Number of observations	550	496	550	496

Notes: Coefficients of linear probability models are shown with robust standard errors in parentheses. Overweight/obese: BMI ≥ 25 kg/m²; Pre-diabetic: FBG (in mmol/L) ≥ 5.6; Pre-hypertensive: SBP/DBP (in mmHg) ≥ 120/80; Metabolic syndrome (MetS): defined through three parameters: waist circumference (in cm) F/M > 80 /94 plus SBP/DBP (in mmHg) ≥ 130 / ≥ 85 and FBG (in mmol/L) ≥ 5.6. Not all control variables are shown for brevity. Included control variables are the same as in all other models: expenditure, education, intensive work, physical activity, age, distance to hospital, being female, being married, household size, smoking, history of diabetes, and history of heart attack. DBP, diastolic blood pressure; FBG, fasting blood glucose; MetS, metabolic syndrome; SBP, systolic blood pressure * Significant at 10% level; ** Significant at 5% level; *** Significant at 1% level.
