# Supplementary material for: Supermarket purchase contributes to nutrition-related non-communicable diseases in urban Kenya
Source: PLoS One. 2017 Sep 21;12(9):e0185148. doi: 10.1371/journal.pone.0185148 (PMC5608323; doi:10.1371/journal.pone.0185148)
Supplement: S4 Table — (PDF) [file pone.0185148.s004.pdf]

S4 Table. Regression results for the effects of supermarkets on BMI, fasting blood glucose, systolic and diastolic blood pressure comparing OLS and IV estimations

	BMI (kg/m²)		FBG (mmol/L)		SBP (mmHg)		DBP (mmHg)	
	OLS	IV	OLS	IV	OLS	IV	OLS	IV
Buys in supermarket	1.15** (0.18)	1.82*** (0.24)	0.16 (0.12)	0.30*** (0.06)	-1.20 (1.03)	1.98 (1.33)	-0.23 (0.54)	1.23 (0.86)
Expenditure per capita	0.11* (0.03)	0.11*** (0.02)	0.01* (0.00)	0.01*** (0.00)	-0.00 (0.04)	-0.03 (0.05)	0.04 (0.05)	0.03 (0.04)
Education, y	0.01 (0.12)	-0.00 (0.10)	-0.00 (0.02)	-0.01 (0.01)	-0.36 (0.20)	-0.42*** (0.14)	-0.18 (0.14)	-0.21** (0.10)
Intensive work, h/wk	0.01 (0.00)	0.01** (0.00)	0.00 (0.00)	0.00 (0.00)	0.00 (0.01)	0.00 (0.01)	-0.00 (0.00)	-0.00 (0.00)
Physical activity, h/wk	-0.02 (0.01)	-0.02** (0.01)	0.00 (0.01)	0.00 (0.00)	-0.02 (0.03)	-0.01 (0.02)	-0.01 (0.01)	-0.01 (0.01)
Age, y	0.11* (0.04)	0.11*** (0.03)	0.02** (0.01)	0.02*** (0.00)	0.87*** (0.03)	0.88*** (0.02)	0.40*** (0.02)	0.41*** (0.02)
Distance to hospital, km	0.04 (0.02)	0.05*** (0.00)	0.02** (0.00)	0.02*** (0.00)	-0.15* (0.04)	-0.09 (0.10)	-0.02 (0.04)	0.01 (0.07)
Female	3.68*** (0.30)	3.59*** (0.28)	0.22 (0.11)	0.20** (0.09)	-4.38 (3.08)	-4.84** (2.31)	-2.60 (1.84)	-2.81** (1.39)
Married	1.04 (0.50)	1.01** (0.45)	-0.11 (0.16)	-0.11 (0.13)	0.08 (1.85)	-0.04 (1.41)	0.61 (0.66)	0.56 (0.51)
Household size	-0.13 (0.05)	-0.12*** (0.04)	-0.01 (0.05)	-0.01 (0.04)	-1.28* (0.35)	-1.21*** (0.25)	-0.57** (0.11)	-0.54*** (0.09)
Smoking	-2.09 (0.79)	-2.14*** (0.65)	-0.16 (0.18)	-0.17 (0.14)	-12.31** (1.88)	-12.57*** (1.40)	-7.18* (2.25)	-7.30*** (1.78)
History diabetes			0.27 (0.19)	0.26* (0.14)				
History heart attack					-0.72 (1.09)	-0.08 (0.36)	-0.79 (2.60)	-0.49 (1.94)
Constant	15.71** (2.60)	15.31*** (2.15)	3.53*** (0.23)	3.46*** (0.19)	114.64*** (6.65)	112.80*** (5.62)	77.57*** (3.21)	76.73*** (2.92)
R-squared	0.23	0.23	0.07	0.07	0.28	0.28	0.18	0.17
Durbin-Wu-Hausman	2.44		4.37		3.80		4.10	
Number of observations	550	550	496	496	550	550	550	550

Notes: Coefficient estimates of OLS and IV models are shown with standard errors in parentheses. Standard errors are cluster-corrected at town level. In the IV regressions, "distance to nearest supermarket" was used as instrument for "buys in supermarket". BMI, body mass index; DBP, diastolic blood pressure; FBG, fasting blood glucose; IV, instrumental variable model; OLS, ordinary least squares; SBP, systolic blood pressure. * Significant at 10% level; ** Significant at 5% level; *** Significant at 1% level.
