# Supplementary material for: Supermarket purchase contributes to nutrition-related non-communicable diseases in urban Kenya
Source: PLoS One. 2017 Sep 21;12(9):e0185148. doi: 10.1371/journal.pone.0185148 (PMC5608323; doi:10.1371/journal.pone.0185148)
Supplement: S5 Table — (PDF) [file pone.0185148.s005.pdf]

S5 Table. Regression results for the effects of supermarkets on the probability of being overweight/obese, pre-diabetic, pre-hypertensive, and suffering from metabolic syndrome comparing OLS and IV estimations

	Overweight/Obese		Pre-diabetic		Pre-hypertensive		MetS	
	OLS	IV	OLS	IV	OLS	IV	OLS	IV
Buys in supermarket	0.119* (0.03)	0.204*** (0.02)	0.108** (0.02)	0.164*** (0.01)	0.006 (0.04)	-0.014 (0.02)	0.048** (0.01)	0.068*** (0.01)
Expenditure per capita	0.008 (0.00)	0.008*** (0.00)	0.002 (0.00)	0.001 (0.00)	-0.001 (0.00)	-0.000 (0.00)	0.000 (0.00)	0.000 (0.00)
Education, y	0.015 (0.01)	0.014* (0.01)	0.000 (0.00)	-0.001 (0.00)	-0.002 (0.01)	-0.001 (0.00)	-0.005 (0.00)	-0.006** (0.00)
Intensive work, h/wk	0.001 (0.00)	0.001** (0.00)	0.000 (0.00)	0.000 (0.00)	-0.000 (0.00)	-0.000 (0.00)	0.000 (0.00)	0.000 (0.00)
Physical activity, h/wk	-0.002 (0.00)	-0.001 (0.00)	0.001 (0.00)	0.001 (0.00)	0.001 (0.00)	0.001 (0.00)	0.000 (0.00)	0.000 (0.00)
Age, y	0.010* (0.00)	0.010*** (0.00)	0.006** (0.00)	0.006*** (0.00)	0.006* (0.00)	0.006*** (0.00)	0.005** (0.00)	0.005*** (0.00)
Distance to hospital, km	0.004 (0.00)	0.005*** (0.00)	-0.000 (0.00)	0.001* (0.00)	-0.003 (0.00)	-0.003*** (0.00)	0.000 (0.00)	0.001*** (0.00)
Female	0.270** (0.05)	0.258*** (0.04)	0.014 (0.02)	0.008 (0.01)	-0.053 (0.03)	-0.050*** (0.02)	0.019 (0.02)	0.017 (0.02)
Married	0.080 (0.06)	0.077 (0.05)	0.025** (0.00)	0.021*** (0.01)	-0.035 (0.02)	-0.034** (0.02)	0.042 (0.04)	0.041 (0.03)
Household size	-0.007 (0.01)	-0.005 (0.01)	0.003 (0.01)	0.004 (0.01)	-0.012 (0.01)	-0.013 (0.01)	-0.002 (0.00)	-0.001 (0.00)
Smoking	-0.197** (0.03)	-0.204*** (0.03)	0.038 (0.02)	0.034*** (0.01)	-0.004 (0.03)	-0.002 (0.03)	-0.048 (0.02)	-0.050*** (0.02)
History diabetes			0.097 (0.05)	0.096** (0.04)				
History heart attack					0.109* (0.03)	0.105*** (0.03)		
History diabetes/heart attack							0.070*** (0.01)	0.071*** (0.01)
Constant	-0.487 (0.20)	-0.537*** (0.16)	-0.258 (0.14)	-0.289** (0.12)	0.764*** (0.04)	0.776*** (0.04)	-0.162* (0.04)	-0.172*** (0.03)
R-squared	0.18	0.18	0.08	0.07	0.05	0.05	0.08	0.08
Durbin-Wu-Hausman	2.42		9.48*		0.12		1.13	
Number of observations	550	550	496	496	550	550	496	496

Notes: Coefficient estimates of linear probability models estimated with OLS and IV are shown with standard errors in parentheses. Standard errors are cluster-corrected at town level. In the IV regressions, “distance to nearest supermarket” was used as instrument for “buys in supermarket”. Overweight/obese: BMI ≥ 25 kg/m²; Pre-diabetic: FBG (in mmol/L) ≥ 5.6; Pre-hypertensive: SBP/DBP (in mmHg) ≥ 120/80; Metabolic syndrome (MetS): defined through three parameters: waist circumference (in cm) F/M > 80 /94 plus SBP/DBP (in mmHg) ≥ 130 / ≥ 85 and FBG (in mmol/L) ≥ 5.6. DBP, diastolic blood pressure; FBG, fasting blood glucose; IV, instrumental variable; OLS, ordinary least squares; MetS, metabolic syndrome; SBP, systolic blood pressure * Significant at 10% level; ** Significant at 5% level; *** Significant at 1% level.
