# Supplementary material for: Supermarket purchase contributes to nutrition-related non-communicable diseases in urban Kenya
Source: PLoS One. 2017 Sep 21;12(9):e0185148. doi: 10.1371/journal.pone.0185148 (PMC5608323; doi:10.1371/journal.pone.0185148)
Supplement: S6 Table — (PDF) [file pone.0185148.s006.pdf]

S6 Table. Regression results for the effects of supermarkets on the probability of being overweight/obese, pre-diabetic, pre-hypertensive, and suffering from metabolic syndrome comparing probit and IV probit estimations

	Overweight/obese		Pre-diabetic		Pre-hypertensive		MetS	
	Probit	IV probit	Probit	IV probit	Probit	IV probit	Probit	IV probit
Buys in supermarket	0.114*** (0.03)	0.112*** (0.02)	0.116*** (0.03)	0.138*** (0.01)	0.003 (0.03)	-0.085*** (0.03)	0.061*** (0.01)	0.055*** (0.02)
Expenditure per capita	0.010*** (0.00)	0.009*** (0.00)	0.002 (0.00)	0.001*** (0.00)	-0.001 (0.00)	0.000 (0.00)	0.000 (0.00)	-0.000 (0.00)
Education, y	0.013* (0.01)	0.012 (0.01)	0.000 (0.00)	-0.001 (0.00)	-0.003 (0.01)	0.000 (0.01)	-0.005* (0.00)	-0.004 (0.00)
Intensive work, h/wk	0.001* (0.00)	0.001* (0.00)	0.000 (0.00)	0.000 (0.00)	-0.000* (0.00)	-0.000** (0.00)	0.000 (0.00)	0.000 (0.00)
Physical activity, h/wk	-0.002 (0.00)	-0.002 (0.00)	0.001 (0.00)	0.001 (0.00)	0.000 (0.00)	0.000 (0.00)	0.000 (0.00)	0.000 (0.00)
Age, y	0.010*** (0.00)	0.010*** (0.00)	0.006*** (0.00)	0.006*** (0.00)	0.008*** (0.00)	0.008*** (0.00)	0.005*** (0.00)	0.005*** (0.00)
Distance to hospital, km	0.003 (0.00)	0.004** (0.00)	0.000 (0.00)	0.002*** (0.00)	-0.003*** (0.00)	-0.006*** (0.00)	0.001*** (0.00)	0.001*** (0.00)
Female	0.275*** (0.04)	0.273*** (0.05)	0.017 (0.02)	0.011 (0.02)	-0.051** (0.02)	-0.037*** (0.01)	0.031 (0.03)	0.034 (0.03)
Married	0.087 (0.06)	0.076 (0.07)	0.032*** (0.01)	0.020 (0.02)	-0.045* (0.02)	-0.033 (0.02)	0.067* (0.03)	0.062** (0.03)
Household size	-0.006 (0.01)	-0.007 (0.01)	0.001 (0.01)	0.002 (0.01)	-0.018* (0.01)	-0.020* (0.01)	-0.002 (0.00)	-0.001 (0.00)
Smoking	-0.200*** (0.02)	-0.204*** (0.02)	0.027 (0.02)	0.035 (0.03)	-0.002 (0.04)	-0.003 (0.04)	-0.052* (0.03)	-0.051** (0.02)
History diabetes			0.083** (0.03)	0.083** (0.03)				
History heart attack					0.159* (0.08)	0.137 (0.10)		
History diabetes/heart attack							0.062*** (0.01)	0.062*** (0.00)
Pseudo R^2	0.15		0.09		0.07		0.18	
Wald statistics	4.36**		4.07**		0.34		1.86	
Number of observations	550	550	496	496	550	550	496	496

Notes: Marginal effects are shown with standard errors in parentheses. Standard errors are cluster-corrected at town level. In the IV probit models, "distance to nearest supermarket" was used as instrument for "buys in supermarket". Overweight/obese: BMI ≥ 25 kg/m²; Pre-diabetic: FBG (in mmol/L) ≥ 5.6; Pre-hypertensive: SBP/DBP (in mmHg) ≥ 120/80; Metabolic syndrome (MetS): defined through three parameters: waist circumference (in cm) F/M > 80 /94 plus SBP/DBP (in mmHg) ≥ 130 / ≥ 85 and FBG (in mmol/L) ≥ 5.6. DBP, diastolic blood pressure; FBG, fasting blood glucose; IV, instrumental variable; MetS, metabolic syndrome; SBP, systolic blood pressure * Significant at 10% level; ** Significant at 5% level; *** Significant at 1% level.
