# Supplementary material for: Supermarket purchase contributes to nutrition-related non-communicable diseases in urban Kenya
Source: PLoS One. 2017 Sep 21;12(9):e0185148. doi: 10.1371/journal.pone.0185148 (PMC5608323; doi:10.1371/journal.pone.0185148)
Supplement: S7 Table — (PDF) [file pone.0185148.s007.pdf]

S7 Table. Full regression results for the effects of supermarket purchase (%) on BMI, fasting blood glucose, systolic and diastolic blood pressure

	BMI (kg/m²)	FBG (mmol/L)	SBP (mmHg)	DBP (mmHg)
Share of supermarket purchase, %	0.15*** (0.02)	0.02*** (0.00)	0.16 (0.11)	0.10 (0.07)
Expenditure per capita	0.09* (0.04)	0.01 (0.00)	-0.05 (0.05)	0.02 (0.04)
Education, y	-0.00 (0.11)	-0.01 (0.01)	-0.43** (0.15)	-0.21 (0.11)
Intensive work, h/wk	0.01* (0.00)	0.00 (0.00)	0.00 (0.01)	-0.00 (0.00)
Physical activity, h/wk	-0.03* (0.01)	-0.00 (0.00)	-0.02 (0.03)	-0.01*** (0.00)
Age, y	0.12*** (0.03)	0.03*** (0.00)	0.89*** (0.02)	0.41*** (0.02)
Distance to hospital, km	0.12*** (0.01)	0.03*** (0.01)	-0.02 (0.15)	0.05 (0.10)
Female	3.80*** (0.37)	0.24*** (0.06)	-4.61 (2.41)	-2.67 (1.45)
Married	0.95* (0.47)	-0.12 (0.12)	-0.10 (1.43)	0.52 (0.50)
Household size	-0.08 (0.07)	-0.00 (0.04)	-1.17*** (0.28)	-0.51*** (0.11)
Smoking	-2.07*** (0.60)	-0.15 (0.12)	-12.49*** (1.37)	-7.25*** (1.78)
History diabetes		0.29 (0.18)		
History heart attack			-0.05 (0.62)	-0.47 (2.03)
Constant	14.22*** (2.18)	3.30*** (0.21)	111.61*** (6.34)	75.99*** (3.32)
Number of observations	550	496	550	550

Notes: Coefficient estimates of instrumental variable models are shown with standard errors in parentheses. Standard errors are cluster-corrected at town level. “Distance to nearest supermarket” was used as instrument for “supermarket purchase”. DBP, diastolic blood pressure; FBG, fasting blood glucose; SBP, systolic blood pressure. * Significant at 10% level; ** Significant at 5% level; *** Significant at 1% level.
