# Supplementary material for: Supermarket purchase contributes to nutrition-related non-communicable diseases in urban Kenya
Source: PLoS One. 2017 Sep 21;12(9):e0185148. doi: 10.1371/journal.pone.0185148 (PMC5608323; doi:10.1371/journal.pone.0185148)
Supplement: S8 Table — (PDF) [file pone.0185148.s008.pdf]

S8 Table. Full regression results for the effects of supermarket purchase (%) on the probability of being overweight/obese, pre-diabetic, pre-hypertensive, and suffering from metabolic syndrome

	Overweight/ Obese	Pre-diabetic	Pre-hypertensive	MetS
Share of supermarket purchase, %	0.016*** (0.00)	0.013*** (0.00)	-0.001 (0.00)	0.005*** (0.00)
Expenditure per capita	0.006 (0.00)	-0.000 (0.00)	-0.000 (0.00)	-0.000 (0.00)
Education, y	0.013* (0.01)	-0.001 (0.00)	-0.001 (0.00)	-0.006** (0.00)
Intensive work, h/wk	0.001** (0.00)	0.000 (0.00)	-0.000 (0.00)	0.000 (0.00)
Physical activity, h/wk	-0.003*** (0.00)	0.000 (0.00)	0.001 (0.00)	-0.000 (0.00)
Age, y	0.011*** (0.00)	0.007*** (0.00)	0.006*** (0.00)	0.005*** (0.00)
Distance to hospital, km	0.013*** (0.00)	0.007*** (0.00)	-0.004** (0.00)	0.003*** (0.00)
Female	0.282*** (0.05)	0.026 (0.02)	-0.051*** (0.02)	0.024 (0.02)
Married	0.070 (0.05)	0.020*** (0.01)	-0.034** (0.02)	0.041 (0.03)
Household size	-0.001 (0.01)	0.007 (0.01)	-0.013 (0.01)	-0.000 (0.00)
Smoking	-0.195*** (0.04)	0.045** (0.02)	-0.003 (0.03)	-0.045*** (0.01)
History diabetes		0.113** (0.05)		
History heart attack			0.104*** (0.03)	
History diabetes/heart attack				0.077*** (0.00)
Constant	-0.660*** (0.16)	-0.379*** (0.13)	0.784*** (0.05)	-0.209*** (0.03)
Number of observations	550	496	550	496

Notes: Coefficient estimates of instrumental variable linear probability models are shown with standard errors in parentheses. Standard errors are cluster-corrected at town level. "Distance to nearest supermarket" was used as instrument for "supermarket purchase". MetS, metabolic syndrome. * Significant at 10% level; ** Significant at 5% level; *** Significant at 1% level.
