# Supplementary material for: Supermarket purchase contributes to nutrition-related non-communicable diseases in urban Kenya
Source: PLoS One. 2017 Sep 21;12(9):e0185148. doi: 10.1371/journal.pone.0185148 (PMC5608323; doi:10.1371/journal.pone.0185148)
Supplement: S9 Table — (PDF) [file pone.0185148.s009.pdf]

S9 Table. Regression results for the effects of supermarkets on BMI with panel data model

	BMI (kg/m²)	
	Fixed effects	Random effects
Buys in supermarket	0.59* (0.34)	0.63** (0.28)
Expenditure per capita, deflated [a]	-0.02 (0.02)	0.06*** (0.01)
Physical activity, h/wk	-0.03*** (0.01)	-0.02*** (0.01)
Age, y	-0.02 (0.04)	0.10*** (0.01)
Female		3.40*** (0.33)
Married	1.02** (0.51)	1.00*** (0.29)
Ol Kalou		-0.75** (0.38)
Njabini		-0.78* (0.42)
Year 2015	0.37** (0.19)	-0.04 (0.13)
Constant	25.51*** (1.50)	18.37*** (0.69)
Wald-chi2		224.91***
F-value	3.58***	
Hausman test	54.47***	
Number of observations	1161	1161

Notes: Coefficient estimates of fixed effects and random effects panel data models are shown with standard errors in parentheses. Hausman test was performed in order to see significant differences between fixed and random effects. Total number of observations for the unbalanced panel data set is 1161 adults (>18 y), including 611 from 2012 and 550 from 2015. [a] 2015 expenditures were adjusted for inflation using official consumer price indices. * Significant at 10% level; ** Significant at 5% level; *** Significant at 1% level.
